# Re-examining the nomenclature of congenital failure of formation in the upper limb: a historical perspective

**DOI:** 10.1177/17531934231160400

**Published:** 2023-03-16

**Authors:** Claudia C. H. Chan, Pauline McGee, Geoffrey Hooper, Wee Leon Lam

**Affiliations:** 1 School of Medicine, University of Edinburgh, Chancellor's Building, Edinburgh, UK; 2 Plastic Surgery Department, Royal Hospital for Children and Young People, Edinburgh; 3 St John's Hospital, Livingston, West Lothian, UK; 4 Retired, St John's Hospital, Livingston, West Lothian, UK

**Keywords:** Congenital hand, phocomelia, Oberg Manske and Tonkin, OMT, thalidomide

## Abstract

In this study, we studied historical case notes to examine nomenclature of congenital upper limb anomalies and explore the changes in terminologies over time. Original diagnoses were reclassified according to previously published classifications and the most recent Oberg, Manske and Tonkin system. Two hundred and thirty-eight case notes were obtained from the period 1961–1991. Hand plate malformations where the diagnosis was obvious or traumatic defects, were excluded. Eighty-six cases (106 extremities) were finally included where an ambiguous diagnosis, such as ‘congenital absence’ was initially given. None of the re-classifications matched the original diagnoses except for cleft hand and radial dysplasia (*n* = 31). Eighteen phocomelia-type limbs were re-classifiable when seen as a continuum of longitudinal deficiency, but not as an intercalary deficit. This study provided further insights into the evolving nature of nomenclature in congenital upper limb anomalies, especially for the condition of phocomelia.

**Level of evidence:** IV

## Introduction

Malformations of the proximal upper limb can occur at several levels and present as a spectrum of congenital upper limb anomalies (CULA) including transverse arrest, longitudinal deficiencies, symbrachydactyly or phocomelia. The nomenclature for such conditions is often debated and is still evolving with the increasing knowledge of upper limb embryology and developmental biology.

O’Rahilly (1951) first proposed a simple classification system for long bone deficiencies, setting the groundwork for classifications of CULAs. The system focused on whether the affected limb displayed a total absence of structure distal to a point (terminal defect) or whether a normal distal structure was present due to a segmental defect (intercalary defect). Frantz and O’Rahilly (1961) further proposed that limb deficiencies can be subclassified as transverse or longitudinal defects depending on where the predominant deficiencies lie. This new classification system was one of the first to be widely accepted and pioneered the use of axial-based classification. Significantly, they grouped the transverse intercalary defects into three types of phocomelia: complete (hand to trunk), proximal (forearm to trunk without a humerus) and distal (hand to humerus without a forearm).

Seemingly straightforward, this subclassification of intercalary defect into three types of phocomelia was subsequently found to be problematic in clinical practice. Tytherleigh-Strong and Hooper (2003) evaluated 44 phocomelic extremities and were only able to classify 11 extremities using the system by Frantz and O’Rahilly (1961). Goldfarb et al. (2005) conducted a similar re-evaluation of 60 limbs previously diagnosed as upper extremity phocomelia and concluded that 49 of these could be re-classified as a continuum of radial or ulnar longitudinal dysplasia rather than true intercalary deficiencies.

Despite these studies, the term ‘phocomelia’ remains firmly entrenched in CULA terminology. In the current International Federation for Societies for Surgery of the Hand (IFSSH) classification of CULAs, otherwise known as the Oberg, Manske and Tonkin (OMT) system, phocomelia continues to be listed as a malformation of the entire upper limb affecting the proximal-distal axis, divided into three sub-groups: proximal (rhizomelic), distal (mesomelic) and proximal + distal (hand to thorax).

A recent opportunity has arisen for the study of historical medical notes of patients, who presented with CULAs under the care of the late Douglas Lamb (Robins et al., 2002). The aim of this study is to examine the original diagnoses given to patients in the 1960–1970s, before the publication of detailed nomenclature studies for conditions such as phocomelia. The secondary aim is to reclassify these diagnoses according to later systems, to investigate how diagnoses have evolved and to offer insights on the future of classification of CULAs.

## Methods

### Case notes study

A retrospective descriptive study was conducted using historical case notes of 238 patients from the archives of a single tertiary congenital hand and amputee clinic at the now defunct Princess Margaret Rose Orthopaedic Hospital, Edinburgh. As this was a historical case-note study, ethical approval was obtained for the secure handling of data and storage of information and no patient consent was required. All patients were treated under the care of Mr Douglas Lamb from May 1961 to September 1991 inclusive. Case notes containing historical photographs, surgical outpatient annotation and operation notes were included in the study, and the notes scanned onto a secure electronic database. Patient demographics, including the date of referral, initial clinical diagnosis and treatment, were extracted from paper notes and entered onto the database. For the purpose of this study, all patients given an initial diagnosis of ‘congenital absence’, ‘congenital amputation’, ‘congenital anomaly’, ‘congenital transverse absence’, ‘congenital transverse defect’, ‘longitudinal upper limb deficiencies’, ‘phocomelia’, ‘radial club hand’ or ‘thalidomide deformities’ were studied, but only those with sufficient medical and photographic records were eligible for inclusion. Patients with conditions that were not congenital in nature or those with incomplete records were excluded from the analysis. In addition, any patients with obvious diagnoses and who had the correct terminologies in their initial diagnoses, such as syndactyly, thumb hypoplasia or clinodactyly, were also excluded.

### Analysis of diagnoses

The IFSSH/OMT system classified CULAs into four main groups: malformations, deformations, dysplasias and syndromes (Goldfarb et al., 2020). For the malformation group, conditions are further divided into those affecting the entire upper limb (early patterning, IA) and hand plate only (late patterning, IB). From our cohort, the diagnoses of patients were reclassified according to the current OMT system and matched against their initial diagnoses by two of the authors (WLL and PM), both consultant hand surgeons with a special interest in congenital hand surgery.

### Further evaluation of malformation: entire upper limb with absent hand

**Figure 1.**
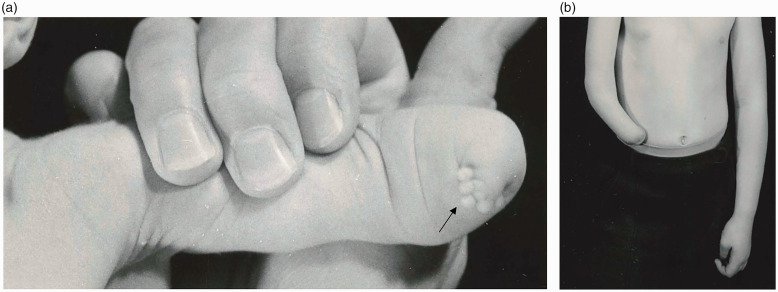
Examples of malformation of the upper limb with hand absent. In this category, upper limbs are either proximal symbrachydactyly with ectodermal elements (a) or transverse arrest (b).

By definition, this group included conditions like symbrachydactyly (Figure 1(a)) and transverse arrest (Figure 1(b)). The presence of ectodermal elements or nubbins were given a diagnosis of symbrachydactyly, whereas a diagnosis of transverse arrest was given in their absence (Goldfarb et al., 2020).

### Further evaluation of malformation: entire upper limb with hand present

By definition, the hand is always present in a patient with phocomelia, although it is often hypoplastic. A further sub-analysis of patients with possible phocomelia was performed using the original Frantz and O’Rahilly (1961) system, and then the Goldfarb et al. (2005) system (Table 1). This group also included non-phocomelic CULAs, such as radial longitudinal deficiencies, as these conditions may overlap with distal phocomelic conditions.

**Table 1.** Classifications of phocomelia.

System	Description
*Frantz and O’Rahilly (*1961 *)*	
Complete phocomelia (Type I*)	Hand-on-thorax
Proximal phocomelia (Type II)	Hand and forearm attached directly to trunk (missing humerus)
Distal phocomelia (Type III**)	Hand attached directly to arm (missing forearm)
*Goldfarb et al. (*2005 *)*	
Proximal radial longitudinal dysplasia	Proximal upper-limb dysplasia characterized by an absent glenoid and absent proximal part of the humerus and distal features of an ulna
Proximal ulnar longitudinal dysplasia	Hypoplastic glenoid with a single arm or forearm bone, with proximal features of a humerus and distal features of a humerus and radius
Severe combined dysplasia (Type A or B)	Type A: distal phocomelia (Type III intercalary deficit**) (missing forearm) Type B: complete phocomelia (Type I intercalary deficit*, hand-on-thorax)
*OMT classification (Goldfarb et al., *2020*)*	
Proximal (humeral: rhizomelic)	Hand and forearm attached to shortened humerus
Distal (forearm: mesomelic)	Hand attached to a shortened forearm with humerus present
Proximal plus distal	Hand-on-thorax

** and *: refer to Frantz and O’Rahilly classification.

### Further evaluation of malformation: hand plate only

In this group, conditions that affected the hand distal to the wrist joint were included. This included conditions like distal symbrachydactyly, transverse arrest and distal ulnar longitudinal deficiency (missing digits). The cleft hand was included due to some ambiguities in the initial described diagnoses when examining the notes. As mentioned, any other patients with obvious hand plate conditions, but who were given the correct diagnoses, were excluded.

### Unclassifiable conditions

Any conditions that we were unable to fit into any of the known OMT system were grouped in this category.

## Results

A total of 238 patient records were available for analysis. Of these, 152 patients with correct hand plate diagnoses (*n* = 54), traumatic injuries (*n* = 42) or lacking photographic records (*n* = 56) were excluded. Eighty-six patients eventually met the inclusion criteria. Twenty patients presented with bilateral upper limb differences resulting in 106 limbs being included in the re-classification process (Table 2).

**Table 2.** Comparison of phocomelic upper limb classification using three classification systems.

Patient	Side	Thalidomide patient	Original diagnosis	Morphology	OMT (Goldfarb et al., 2020)	Goldfarb et al. (2005)	Frantz and O’Rahilly (1961)
1	Lt	No	Absence of humerus and radius with two-digit hand	Short humerus with short forearm and two-digit hand	Unclassifiable	Proximal radial ulnar longitudinal deficiency	Unclassifiable
2	Rt	No	Congenital absence	Single digit hypoplastic hand with severely hypoplastic forearm and humeral elements	Unclassifiable	Proximal radial ulnar longitudinal deficiency	Unclassifiable
	Lt	No	Congenital absence	Single digit hypoplastic hand with severely hypoplastic forearm and humeral elements	Unclassifiable	Proximal radial ulnar longitudinal deficiency	Unclassifiable
3	Rt	No	Congenital absence of upper limb	Two-digit hand attached to elbow with no forearm	Distal (I-A-1-iv-b)	Combined severe dysplasia type A	Type III
	Lt	No	Congenital absence of upper limb	Rudimentary hand on thorax	Proximal plus distal (I-A-1-iv-c)	Combined severe dysplasia type B	Type I
4	Lt	Yes	Left rudimentary upper limb	Single digits rudimentary hand attached to trunk with abnormal shoulder	Proximal plus distal (I-A-1-iv-c)	Combined severe dysplasia type B	Type I
5	Rt	No	Phocomelia	Two-digit hand attached to near normal length of humerus with hypoplastic shoulder	Distal (I-A-1-iv-b)	Combined severe dysplasia type A	Type III
6	Lt	No	Phocomelia	Thumb and two-digit hand attached directly to thorax	Proximal plus distal (I-A-1-iv-c)	Combined severe dysplasia type B	Type I
7	Rt	Yes	Phocomelia	Four finger hand, no thumb shortened forearm and humerus	Unclassifiable	Proximal radial ulnar longitudinal deficiency	Unclassifiable
	Lt	Yes	Phocomelia	Four finger hand, no thumb shortened forearm and humerus	Unclassifiable	Proximal radial ulnar longitudinal deficiency	Unclassifiable
8	Rt	No	Phocomelia	Hypoplastic thumb and two-digit hand attached directly to thorax	Proximal plus distal (I-A-1-iv-c)	Combined severe dysplasia type B	Type I
9	Rt	Yes	Thalidomide deformities	Three-digit hand with short humerus and forearm	Unclassifiable	Proximal radial ulnar longitudinal deficiency	Unclassifiable
	Lt	Yes	Thalidomide deformities	Three-digit hand with short humerus and forearm	Unclassifiable	Proximal radial ulnar longitudinal deficiency	Unclassifiable
10	Rt	Yes	Thalidomide deformities	Two-digit hand with short forearm and humerus	Unclassifiable	Proximal radial ulnar longitudinal deficiency	Unclassifiable
	Lt	Yes	Thalidomide deformities	Single digit hand with short forearm and humerus	Unclassifiable	Proximal radial ulnar longitudinal deficiency	Unclassifiable
11	Rt	Yes	Thalidomide deformities	Single digit hypoplastic hand attached directly to thorax	Proximal plus distal (I-A-1-iv-c)	Combined severe dysplasia type B	Type I
12	Rt	Yes	Thalidomide deformities	Rudimentary three-digit hand with hypoplastic thumb, short forearm and humerus	Unclassifiable	Proximal radial ulnar longitudinal deficiency	Unclassifiable
	Lt	Yes	Thalidomide deformities	Two-digit hand with severely shortened forearm and humerus	Unclassifiable	Proximal radial ulnar longitudinal deficiency	Unclassifiable

OMT: Oberg, Manske and Tonkin.

‘Congenital absence’ was the most common original diagnosis given to patients (*n* = 43), followed by ‘radial club hand’ (*n* = 13), ‘congenital amputation’ (*n* = 7), ‘thalidomide deformities’ (*n* = 6), ‘phocomelia’ (*n = *4), ‘congenital deformity’ (*n = *3), ‘congenital abnormalities’ (*n* = 3), ‘congenital transverse defect’ (*n* = 2), ‘syndrome’ (*n* = 2), ‘congenital central defect’ (*n* = 1), ‘congenital terminal defect’ (*n* = 1) and ‘longitudinal upper limb deficiency’ (*n* = 1).

### Malformation: entire upper limb with hand absent

In this category, 37 limbs were reclassified using the OMT system as symbrachydactyly (IA-1-ii) (*n* = 12) or transverse arrest (IA-1-iii) (*n* = 25) (Figure 1). The most common original diagnosis given to patients for these two conditions was ‘congenital absence’ (*n* = 22).

### Malformation (phocomelia type conditions): entire upper limb with hand present

**Figure 2.**
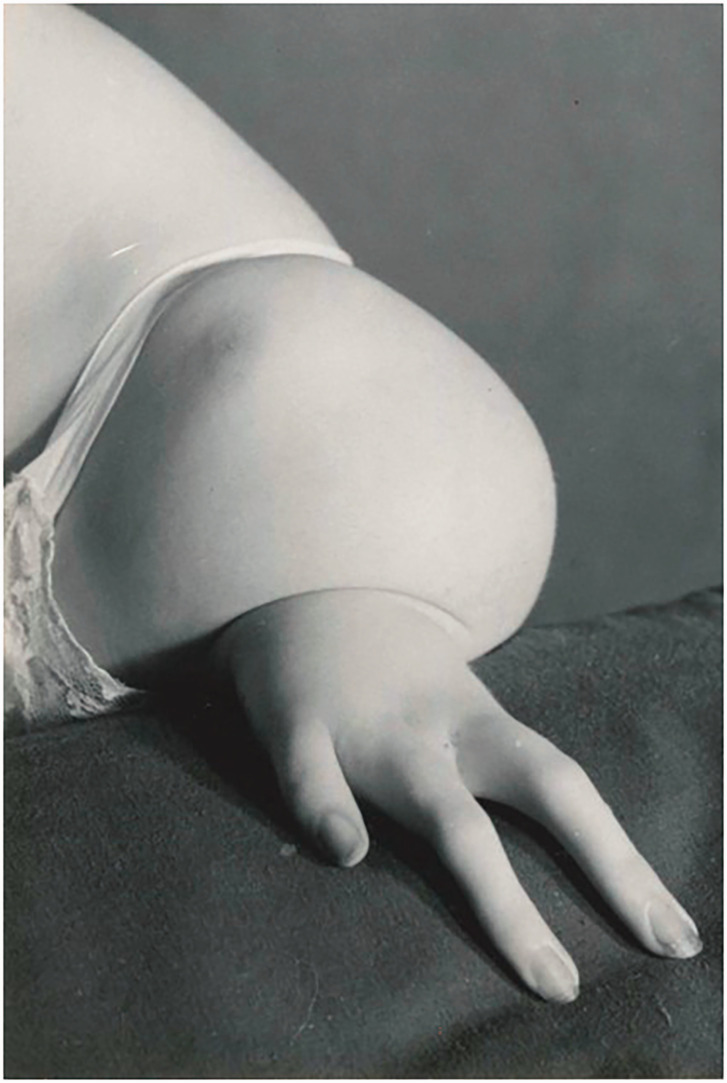
Example of malformation of the upper limb with hand present. A type of proximal radial/ulnar longitudinal deficiency (Goldfarb et al., 2005, classification) characterized by shortened humerus and shortened forearm with a hypoplastic hand.

**Figure 3.**
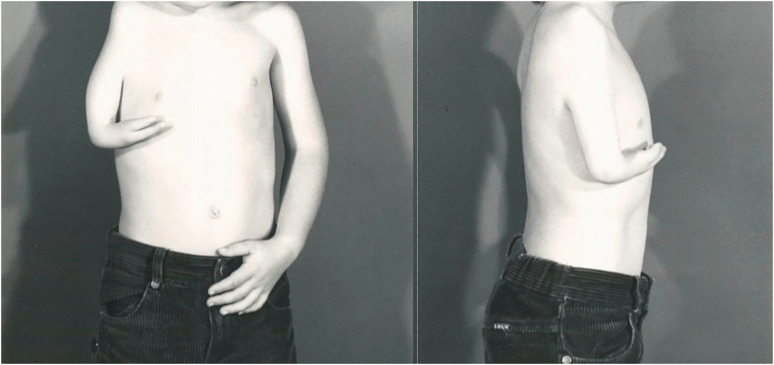
Example of malformation of the upper limb with hand present. A type of severe combined dysplasia Type A characterized by a near normal humerus, missing forearm and a hypoplastic hand.

**Figure 4.**
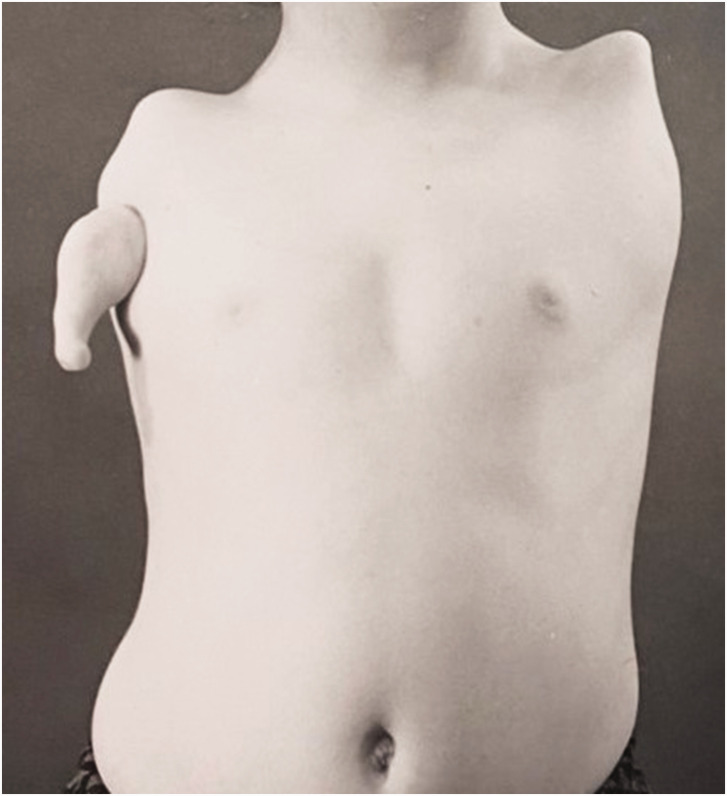
Example of malformation of the upper limb with hand present. A type of severe combined dysplasia Type B characterized by a hand-on-thorax on the right and complete amelia on the left.

Twelve patients with 18 limbs were reclassified as having a type of phocomelia. These were also re-classified according to the systems as described by Frantz and O’Rahilly (1961) and Goldfarb et al. (2005) (Table 2). Only seven of the extremities could be classified using the former, but all extremities were re-classifiable according to the Goldfarb et al. (2005) system. Limited radiographs challenged the evaluation of whether these were proximal radial or ulnar deficiencies, hence 11 extremities were considered to be proximal radial/ulnar longitudinal deficiencies (Figure 2), two were combined severe dysplasia Type A (Figure 3) and five were combined severe dysplasia Type B (Figure 4) according to the Goldfarb et al. (2005) classification. There were seven thalidomide patients in this group with 12 limbs involved.

### Malformation: hand plate only

Fourteen limbs presented with malformations of the hand plate only. None of the reclassifications (I-B) matched the original diagnoses except for the cleft hand (*n* = 13). In the notes, the now discarded term ‘lobster claw hand’ was used instead of cleft hand.

### Unclassifiable conditions

**Figure 5.**
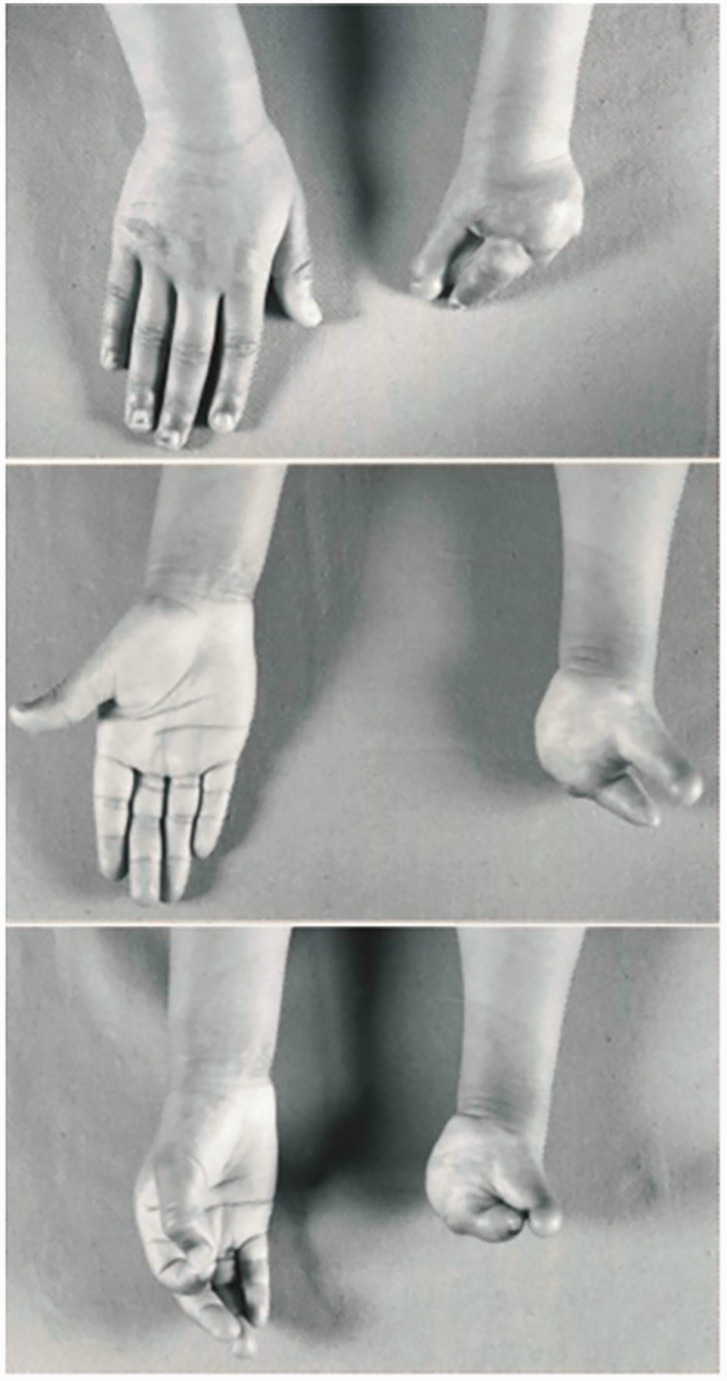
Example of unclassifiable deformity. In this instance, the diagnosis was described as ‘most interesting and unusual deformity …’ and also ‘bizarre and unusual deformity’.

This group consisted of four patients with complex or multiple presentations on one limb that could not be obviously classified to one of the categories described above (Figure 5).

### Radial longitudinal deficiencies

**Figure 6.**
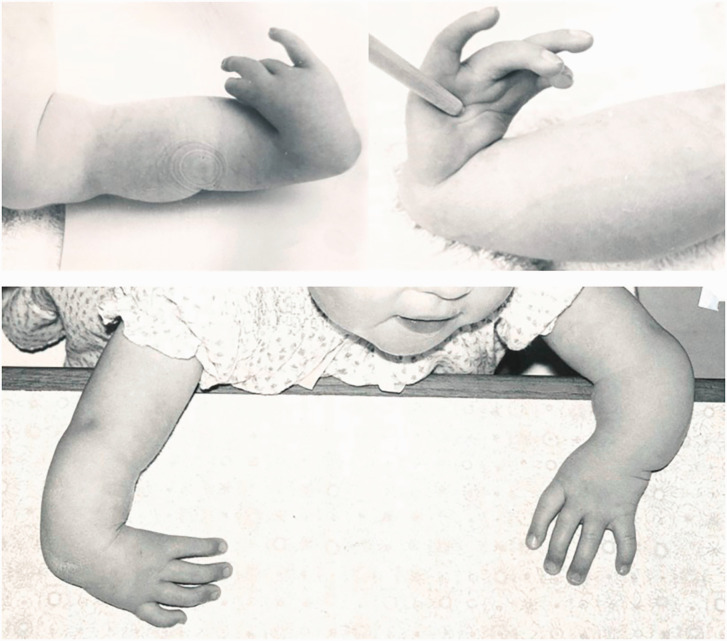
Example of radial longitudinal deficiency. These patients were all diagnosed as ‘radial club hand’.

Thirteen patients were diagnosed as ‘radial club hand’ with 18 limbs involved. The photographs of these patients were examined in detail to ensure these were not a type of proximal phocomelia, which would be considered as such in the system of Goldfarb et al. (2005). All 18 of the ‘radial club hands’ were correctly classified as radial longitudinal deficiencies (Figure 6).

### Thalidomide patients

**Figure 7.**
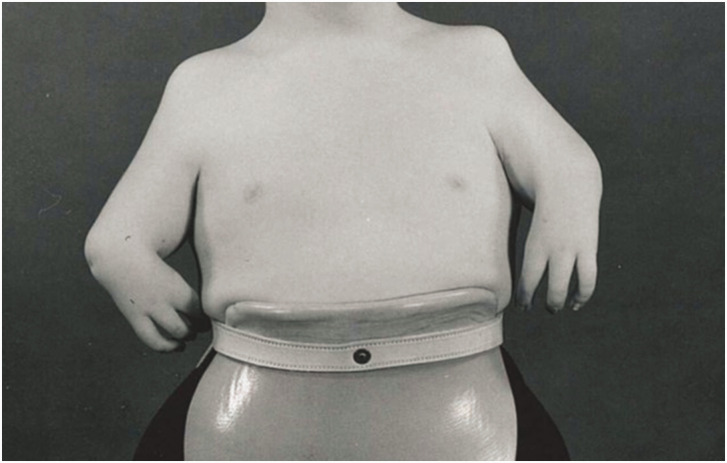
Example of a patient affected by thalidomide embryopathy. In this instance, the descriptions would fit those of a severe combined dysplasia type A bilaterally (Goldfarb et al., 2005, classification).

Patients affected by thalidomide embryopathy presented with a variety of CULAs. Seven thalidomide patients were identified with ten upper limbs affected. The most common diagnoses were ‘thalidomide deformities’ (*n* = 6), followed by ‘longitudinal upper limb deficiencies’ (*n* = 1). Most limbs were reclassified as ‘proximal radial/ulnar dysplasia’ (*n* = 8), using the Goldfarb et al. (2005) system (Figure 7).

## Discussion

In this study, we examined the historical case notes of the late Douglas Lamb to study the evolving nature of nomenclature in diagnosing CULAs. We found that the majority of diagnoses given in the 1960s and 1970s for these patients differ when renamed according to present classification systems like the OMT. We also found that several conditions no longer adhere to the original phocomelia classification proposed by Frantz and O’Rahilly (1961). Finally, with the exception of the cleft hand, none of the hand plate malformations matched their original diagnoses.

Nomenclature for the diagnosis of CULAs remains an evolving subject. The problem is compounded when the nomenclature for CULAs was less defined and clear diagnostic terms were yet to be established, as was the case several decades ago. In our study, we had an opportunity to study historical notes from an interesting era in the 1960s and 1970s when there was a significant incidence of severe upper limb deficiency conditions, coinciding with the thalidomide tragedy (Lamb, 1977). In that same period, the work of Frantz and O’Rahilly (1961) on the classification of phocomelia had just been published. In addition, Swanson (1965) had also just published his article on phocomelia, but it was not until 1976 that he proposed his more comprehensive classification system, which was subsequently adopted by the IFSSH. There was, otherwise, a paucity of literature that would allow agreement in the naming of CULA conditions.

In our review, we found ‘congenital absences’ to be the most common diagnosis given to patients. Most of these patients had a missing hand, with or without distal elements, constituting transverse arrest and symbrachydactyly, respectively. These conditions were not well described in the 1960–1970s; Birch-Jensen (1949) briefly described symbrachydactyly as a unilateral condition occurring sporadically in families, and when described in conjunction with Poland syndrome, these were usually limited to the CULAs of the hand plate.

The challenge continues even in the present with modern classification such as the OMT system, where clear categories and nomenclature are given. The aim of the OMT was to improve on the ambiguity of the Swanson classification by comprehensively naming all CULAs. In the recent study by Sletten et al. (2022), a group of five experienced surgeons tried to group CULAs according to the updated OMT (Goldfarb et al., 2020). While they managed to achieve excellent agreement on the majority of the more common conditions, there were other groups in which consensuses could not be met. For example, they could not agree on what constituted a symbrachydactyly or transverse arrest. This resulted in an overall good inter-rater reliability for common conditions like cutaneous syndactyly, but only moderate reliability for others.

The results of this study by Sletten et al. (2022) revealed a continued challenge in nomenclature for CULA, even in the present day. The issue may not lie in the actual use of the correct nomenclature but elsewhere; for example, in certain conditions, the morphology (appearance) may not always yield a clear diagnosis. Furthermore, surgeons may differ in their opinions even when looking at the same hand. Ultimately, some conditions may require genetic diagnoses for confirmation rather than depending on the phenotypic appearances, for which a degree of subjectivity may always exist.

Another aspect of nomenclature that requires ongoing discussion is in regard to the phocomelic type conditions. Of the 18 limbs reclassified as having a type of phocomelia, only seven could be classified using the original Frantz and O’Rahilly system, but all extremities were re-classifiable according to the Goldfarb et al. (2005) system. Phocomelia refers to an intercalary segmental defect, as opposed to a terminal defect, namely, with an amputation-type stump. Although Swanson (1976) reiterated the classification of Frantz and O’Rahilly (1961) in his article by describing the three types of phocomelia – proximal, distal and complete – he expressed doubts whether true intercalary defects existed. It would appear that he would have preferred to see these conditions as severe forms of longitudinal ‘failure of formation’ or deficiencies, a theme that was picked up by Tytherleigh-Strong and Hooper (2003) and included in the classification by Goldfarb et al. (2005). Despite this, the current OMT continues to include the three sub-types of phocomelia, categorized under defects of the proximal–distal axis affecting the entire upper limb (IA-1-iv-a-c). In the future, an alternative would be to consider these conditions as deficiencies of the humerus and forearm bones by naming them as severe longitudinal deficiencies. However, such conditions would then have to be moved from the proximal–distal axis to the radial–ulnar axis.

The most important limitation of this study is the lack of radiographs as unfortunately, these were not kept with the original notes. Especially with the proximal radial–ulnar deficiency phocomelia-type cases, it was not possible to evaluate whether these were radial or ulnar deficiencies. We have therefore grouped these together as proximal radial/ulnar longitudinal deficiencies. Formal radiology reports were very few in numbers and contained too little information for analysis. The radiological descriptions, if any, were included as a part of the clinic annotations. But again, this did not provide any more information than the proposed diagnosis. As the study focused on a comparison of nomenclature, we felt there was sufficient interesting material to be studied by examining the case notes and photographs, even without radiographs. Another important limitation pertains to the retrospective nature of the study. Our patient population were referred from wider parts of Scotland and the wider United Kingdom, and therefore did not represent the occurrence of these conditions in the Edinburgh population at the time.

In conclusion, this historical case note study exercise has allowed us to observe an interesting evolution of nomenclature for CULAs over the last few decades. It has also allowed us to see that naming of all CULAs may be an ongoing challenge. Differences in nomenclature between surgeons may not be the result of the system or terminologies, but ultimately a result of their subjectivity and experience.
